# Chitosan-Based Hemostatic Hydrogels: The Concept, Mechanism, Application, and Prospects

**DOI:** 10.3390/molecules28031473

**Published:** 2023-02-03

**Authors:** Peng Fan, Yanbo Zeng, Dionisio Zaldivar-Silva, Lissette Agüero, Shige Wang

**Affiliations:** 1 School of Materials and Chemistry, University of Shanghai for Science and Technology, No. 516 Jungong Road, Shanghai 200093, China; 2 Department of Gastroenterology, Changhai Hospital, Naval Medical University, No. 168 Changhai Road, Shanghai 200433, China; 3 USST-UH International Joint Laboratory for Tumor Diagnosis and Energy Treatment, University of Shanghai for Science and Technology, Shanghai 200093, China; 4 Departamento de Biomateriales Poliméricos, Centro de Biomateriales, Universidad de La Habana, Ave. Universidad entre Calle Ronda y Calle G, Municipio Plaza de la Revolución 10400, Cuba

**Keywords:** hemostasis, chitosan, hydrogel, self-healing, chemical modification

## Abstract

The design of new hemostatic materials to mitigate uncontrolled bleeding in emergencies is challenging. Chitosan-based hemostatic hydrogels have frequently been used for hemostasis due to their unique biocompatibility, tunable mechanical properties, injectability, and ease of handling. Moreover, chitosan (CS) absorbs red blood cells and activates platelets to promote hemostasis. Benefiting from these desired properties, the hemostatic application of CS hydrogels is attracting ever-increasing research attention. This paper reviews the recent research progress of CS-based hemostatic hydrogels and their advantageous characteristics compared to traditional hemostatic materials. The effects of the hemostatic mechanism, effects of deacetylation degree, relative molecular mass, and chemical modification on the hemostatic performance of CS hydrogels are summarized. Meanwhile, some typical applications of CS hydrogels are introduced to provide references for the preparation of efficient hemostatic hydrogels. Finally, the future perspectives of CS-based hemostatic hydrogels are presented.

## 1. Introduction

Uncontrolled bleeding caused by trauma can lead to various complications such as hypothermia, decreased blood pressure, bacterial infection, and even shock [1,2]. If not handled in time, it will cause a significant increase in morbidity and mortality [3]. Cardiac, hepatic, and orthopedic surgical procedures are also associated with bleeding and adverse side effects such as hypovolemic shock and hypothermic shock coagulopathy [4]. Therefore, research on the fundamentals of hemostasis and development of effective hemostatic materials have great significance to the saving of lives in emergencies.

Traditional hemostatic materials are usually bandages and gauze dressings, which stop bleeding by direct compression [5,6]. They are easy to manufacture, inexpensive, and reusable. However, in the presence of blood or tissue fluids, traditional hemostatic materials are susceptible to bacterial infection [7]. In addition, the tearing of the dressing may cause pain and prolonged wound healing time [8], which limit their further applications. Moreover, they are not able to adapt to the shapes of irregular, deep, and narrow wounds. Topical hemostatic agents, adhesives, and sealants have been developed over the past few decades and have shown a high potential for hemostasis in surgical and emergencies [9,10,11]. Hemostatic agents are known to stop bleeding by augmenting the blood clotting cascade, while adhesives hold various tissues and blood vessels together. Nevertheless, some disadvantages may limit the general applicability of these products. For example, fibrinogen and thrombin-based fibrin sealants may fail to stop bleeding and cause infection because they lack good adhesive properties and have a tendency to be displaced during the washout of blood [12,13]. Although cyanoacrylates are powerful hemostatic adhesives used to solve these problems, they can cause allergic reactions [14]. Furthermore, cyanoacrylates can produce heat rapidly during the curing process, and the degradation products may be toxic and have adverse effects on humans. Therefore, the development of safe, fast, and efficient hemostasis materials has become an in-demand research topic. 

**Figure 1:**
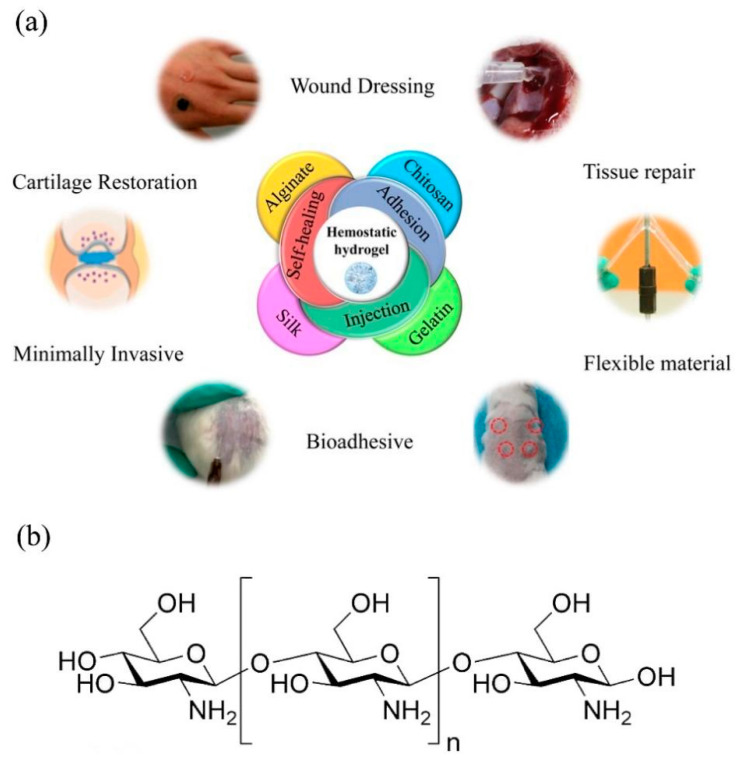
(**a**) Properties and applications of hemostatic hydrogels prepared from different polymers [23,24,25,26,27,28]. Copyright 2019, 2021 Elsevier. Copyright 2022 Taylor & Francis Group. Copyright 2019, 2021 American Chemical Society. (**b**) Chemical structure of CS.

Hydrogels are prepared from natural or synthetic polymers and have promising applications in the field of hemostatic materials [15,16]. Different natural polymers and their derivatives and synthetic polymers that can be processed into hydrogels are shown in Table 1. Typical hydrogels have excellent injectability, which allows them to be easily applied at target sites and fill irregularly shaped wounds through minimally invasive procedures [17,18]. In the case of natural polysaccharides, which are frequently used as the raw materials for the preparation of the hydrogels, their relevant characteristics, such as abundant sources, biodegradation, good biocompatibility, and easy modification, ensure the effective hemostasis and subsequent wound healing [19,20]. An ideal polymer hydrogel for hemostasis is expected to have the following features [21,22]: (1) it should have a rapid gelation rate to stop bleeding instantly and facilitate active wound healing; (2) in dynamic and humid environments, the hemostatic hydrogel should have sufficient adhesion and outstanding mechanical properties to seal the wound and avoid the migration of the hemostatic hydrogel from the bleeding site; and (3) it should have good biocompatibility. In addition, controllable swelling behaviors are necessary, since highly swollen hydrogels may cause the compression of the surrounding tissue. As shown in Figure 1a, different natural or synthetic polymers have been designed to prepare hemostatic hydrogels. 

Chitosan (CS) can be made from chitin by a deacetylation reaction [29]. As shown in Figure 1b, a large number of amino and hydroxyl groups on the CS macromolecular chain provides different modification opportunities of acylation, esterification, carboxylation, acidification, and other reactions [30,31]. With the rapid development of biomaterials science, CS-based hemostatic hydrogels have shown great vitality owing to their good hemostatic effect, biodegradability, antibacterial ability, and healing-promoting properties [32]. Several recent reviews have been published on hemostatic materials. For example, Xia et al. [33] summarized the physiological coagulation process of CS and discussed the advantages and disadvantages of CS hemostatic materials. In another study, Hu et al. [34] reviewed the mechanisms of hemostasis and design principles of hemostatic materials for coagulation disorders and briefly discussed the challenges and prospects of hemostatic materials for coagulation disorders. Simpson et al. [35] reviewed the recent developments in the field of materials for topical hemostatic agents and emergency medicine. However, a systematic summary of the hemostatic mechanism of CS hydrogels—together with a state-of-the-art on the preparation of CS hydrogel and its application in hemostatic materials—have not yet been reported. In this review, we describe the process and principles of hemostasis and summarize the development status, characteristics, and advantages of CS hemostatic hydrogels. Using this foundation, the hemostatic mechanisms, preparation methods, and research progress of the use of CS hydrogels as hemostatic agents are discussed. Finally, the prospective future development of CS-based hemostatic hydrogels is presented.

## 2. Hemostatic Mechanism of CS Hydrogels

### 2.1. The Principle of Hemostasis

Hemostasis, as a complex physiological process, involves the cells, extracellular matrix components, and signaling compounds [53]. It can be generally divided into the following three processes: vasoconstriction or blockage, the formation of platelet blockage, and coagulation or clotting [54]. When bleeding occurs, the blood vessels are ruptured and the body releases thromboxane and epinephrine, which stimulate local and systemic vasoconstriction and reduce the flow and loss of blood [55,56,57]. At the same time, once the blood comes into contact with the collagen in the outer layer of the vascular membrane, the platelets adhere to the collagen because of its adhesive properties [58]. Platelets release chemical messengers (such as diphosphate (ADP) and thromboxane), thus increasing the number of platelets to aggregate at the injury site and further enhancing the vasoconstriction [59]. As a result, a platelet plug is formed to physically prevent the blood from escaping the vessel. After that, the blood clotting mechanism is activated through two main ways: the endogenous coagulation pathway and the exogenous coagulation pathway [60]. 

The coagulation factors involved in hemostasis are indicated by Roman numerals. The endogenous coagulation pathway plays a key role in the maintenance and consolidation phase after the initiation of the coagulation process. The endogenous coagulation is initiated by the activation of factor XII to produce XIIa after foreign body exposure, which in turn activates XI to XIa. Then, XIa activates IXa in the presence of Ca^2+^, which forms a complex with activated VIIIa, PF_3_, and Ca^2+^ to further activate X. The activated partial thromboplastin reaction time is mainly used clinically to reflect the degree of the rapidity of the endogenous coagulation pathway [61]. In the exogenous coagulation pathway, the factors involved in coagulation are both internal plasma factors and external plasma tissue factors. Injured tissue releases the tissue factor (TF, III) which binds to coagulation factor FVII to form a complex. This complex is then activated by coagulation factor FXa, which is converted to FVIIa, resulting in the formation of the VIIa tissue factor complex and the activation of factor X. Clinically, prothrombin reaction time is used to describe the rapidity of exogenous clotting [62]. The hemostasis mechanism of hemostatic agents is divided into three categories. The first one provides external hemostatic substances to increase the concentration of clotting factors at the bleeding site. The second type is through hemostatic agents, which aggregate clotting factors in the bleeding area. The third type is responsible for directly sealing the wound surface using the strong adhesive force of the hemostatic agents [63].

### 2.2. The Hemostatic Mechanism of CS

CS plays an important role in all processes of hemostasis. During hemostasis, CS can induce the aggregation of platelets and plasma proteins to help the blood clotting and vasoconstriction at the injured site [64,65,66,67]. Moreover, CS has been found to enhance the function of polymorphonuclear leukocytes, macrophages, and fibroblasts [68]. In a study, CS was found to promote the production of hyaluronic acid at the injury site, which induced wound healing faster [69]. 

**Figure 2:**
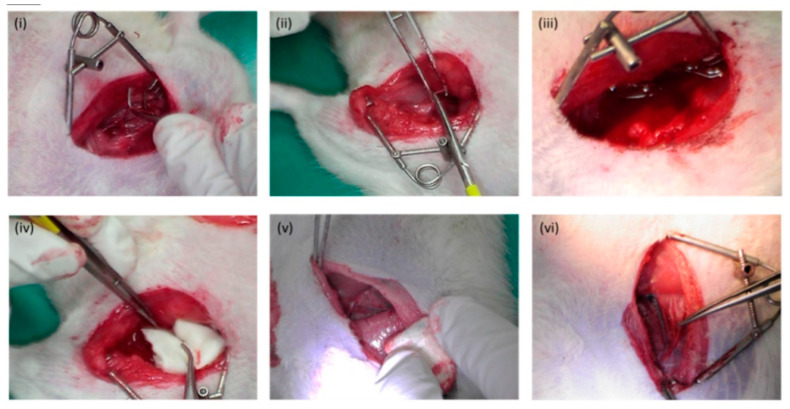
The hemostatic effect of the dressing with a rat femoral artery hemorrhage model. (**i**–**iii**) for compression hemostasis; (**iv**–**vi**) for CS dressing hemostasis [67]. Copyright 2019 Creative Common.

Attracting and gathering red blood cells is the first step in CS to stop bleeding. The CS-acetic acid salt can cause red blood cells to adhere, aggregate, deform, and finally, form a firm blood clot to block the bleeding point. Many hemostatic agents are ineffective or limited in coagulopathies where coagulation is lost [34]. However, the hemostatic mechanism of CS does not depend on the classical coagulation cascade, which makes CS distinctly different from other hemostatic agents [70]. Wang et al. [67] compared the hemostatic potential of CS and gauze. The CS-free gauze was not able to absorb blood adequately. In contrast, CS dressings leave relatively little blood in the wound, meaning that CS absorbs hemoglobin from the blood more effectively than gauze. In order to determine the relative amount of blood that was not absorbed, a hemoglobin assay was performed, which showed that the residual hemoglobin concentration of CS in saline is significantly lower than that of gauze. Additionally, the hemostatic potential of dressings was assessed in a rat femoral artery hemorrhage model. The assessment of the hemostatic abilities of the different materials was performed by measuring the time required to stop the bleeding. Fourteen rats were randomly divided into two equal groups, one with a CS dressing and the other without a CS dressing. The hemostatic time of gauze without a CS dressing was 7.3 ± 1.2 min (Figure 2i,ii,iii), and the hemostatic time of rats in the CS dressing group was 5.4 ± 0.5 min (Figure 2iv,v,vi), indicating that the hemostatic effect of CS was significantly better than that of the control group (*p* = 0.045). This is attributed to the electrostatic interaction between CS and the erythrocytes that form an agglutination network. In addition, CS can also bind to hemoglobin and change its microstructure to form complexes through hydrogen interactions [71]. 

The second step of CS hemostasis is the induction of platelet aggregation and activation [72,73]. After CS contacted the blood, the level of platelet activation markers (platelet factor-4, β-thrombomodulin) increased, and the level of Ca^2+^ in platelets was significantly increased, which elevated the expression of glycoprotein IIb/IIIa (glycoprotein II b/IIIa, GP IIb/IIIa) in the platelet membrane. For example, a sodium tripolyphosphate cross-linked CS-based soft hemostatic hydrogel was synthesized by Patil et al. [74]. CS-based hemostatic hydrogel dressing incorporates hemostasis agonists like aluminum chloride, an aggregation enhancer, and silica nanoparticles, a contact activator. The sodium tripolyphosphate cross-linked CS-based soft hemostatic hydrogel showed enhanced aggregation in platelets compared to SiNPs, AlCl_3_, or CS alone. As a positive control, cells treated with SFLLRN peptide and ADP also showed platelet aggregation. In contrast, this phenomenon did not occur in heparin-treated cells because heparin inhibited the thrombin-mediated platelet aggregation.

Moreover, the physical properties of CS itself endow it with the property of excellent non-specific membrane adhesion. CS can adhere to bleeding wounds and combine with hemoglobin through hydrogen bonds, electrostatic interactions, and hydrophobic interactions to form a CS/hemoglobin complex, resulting in changes in the microstructure of hemoglobin and an increase in viscosity [75]. In addition, after stopping the bleeding, CS forms an antibacterial layer over the bleeding site, which protects it from bacterial infection and promotes wound healing [67].

**Figure 3:**
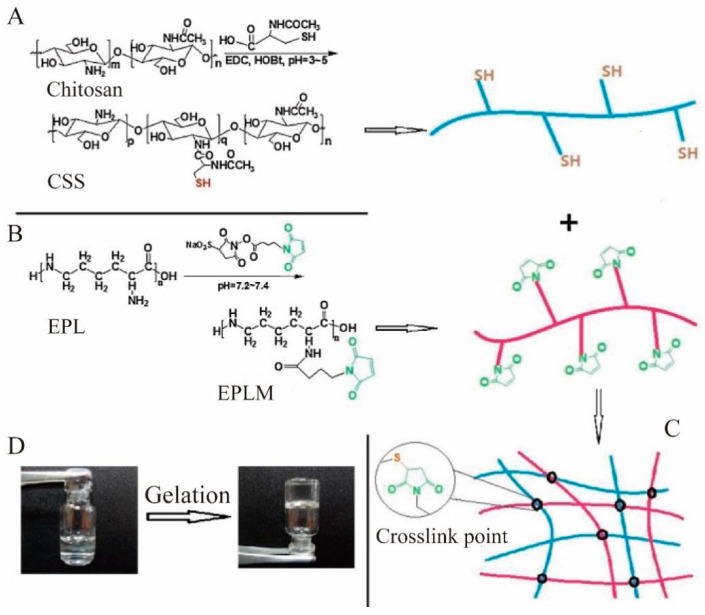
(**A**) Schematic diagram of CSS synthesis; (**B**) schematic diagram of EPLM synthesis; (**C**) in situ hydrogel formation; (**D**) photographs of hydrogel formation [81]. Copyright: 2013 Elsevier.

The hemostatic mechanism of CS derivatives is consistent with CS. For instance, introducing quaternary ammonium groups increases the number of positively charged centers of CS, therefore, the platelet aggregation is higher than in CS. Furthermore, it has been found that platelet aggregation increases with the degree of substitution [76]. However, compared with CS, the incorporation of carboxymethyl CS did not further promote red blood cell aggregation. Nevertheless, due to the enhanced hydrophilicity, the above derivatives can be prepared into a highly absorbent hemostatic product to increase the concentration of local coagulation factors, red blood cells, and platelets and promote the coagulation process [77]. In addition, the introduction of succinyl groups to carboxymethyl chitosan can also shorten the activation time of coagulation proteins. For example, a novel n-succinyl CS (N-SuC) was prepared using an ionic gelation method, and its potential wound healing was tested under in vitro and in vivo conditions. The prepared N-SuC NPs films exhibited significant antibacterial activity against *Escherichia coli* and *Staphylococcus aureus* with minimum inhibitory concentrations of 6 mg/mL and 8 mg/mL, respectively. The biocompatibility and in vitro wound healing activity of N-SuC were investigated using human dermal fibroblasts. It was shown that N-SuC significantly accelerated wound healing by inducing a higher level of angiogenesis and granulation tissue formation [78]. Another interesting modification is related to phosphorylated CS, which can mimic the hemostatic effect of the natural inorganic polymer polyphosphate. The interaction of phosphorylated CS with charged biomaterials was investigated by Sperling et al. [79] The phosphorylation of CS may improve the hemocompatibility of the CS surface, thereby promoting more cations to interact with factor V and factor XI. Furthermore, CS is also able to be modified by sulfhydryl groups, which facilitate the adsorption of red blood cells and platelets, increase the concentration of clotting factors at the bleeding site, and accelerate the hemostasis [80]. As shown in Figure 3, using sulfhydryl-modified CS (CSS) as an efficient peptide cross-linking agent (EPLM), Nie et al. [81] prepared a novel hemostatic hydrogel capable of rapid cross-linking by introducing maleimide groups to hydrolyzed lysine under mild conditions (Figure 3A–C). This hemostatic hydrogel allowed gelation to occur rapidly within 15–215 s by modulating the CSS concentration, degree of substitution of the maleimide group, and other factors (Figure 3D). Furthermore, the hydrogel had four times the adhesive strength of commercial fibrin gels and was used as a hemostatic adhesive. In addition, in experiments conducted to assess hemostatic performance, six rats were used in each experimental group to measure blood loss with and without a hydrogel at the bleeding site. The total blood loss in the untreated liver was approximately 106.7 ± 27 mg in 3 min (sulfhydryl-modified CS group: 28.3 ± 5 mg), suggesting that sulfhydryl-modified CS had a significant hemostatic effect (* *p* < 0.01). In order to assess hemostatic performance, six rats were used in each experimental group to measure blood loss with and without hydrogel at the bleeding site. The total blood loss in the untreated liver was approximately 106.7 ± 27 mg in 3 min compared to 28.3 ± 5 mg in the sulfhydryl-modified CS. Sulfhydryl-modified CS had a significant hemostatic effect (* p < 0.01).

Studies have shown that hemolysis is a major challenge to the preparation of hemocompatible hemostatic materials [82,83,84,85]. Therefore, the hemocompatibility of a hemostatic material is evaluated by the degree of erythrocyte lysis and hemoglobin dissociation. In general, residual and low molecular weight components of the material may react with erythrocyte proteins, disrupt the integrity of erythrocytes, and lead to hemolysis [85]. Chen et al. [86] evaluated the hemolytic properties of the porous carboxymethyl CS-grafted polyacrylic acid. The hemolysis ratio of the unmodified CS leaching solution was 3.1%, while the hemolysis ratio of the modified CS leaching solution was 0.5%. These results indicate that porous polymer is a promising hemostatic wound dressing.

### 2.3. Effect of CS Deacetylation Degree and Relative Molecular Weight on Hemostasis

The hemostatic effect of CS is affected by the degree of deacetylation, relative molecular weight, and chemical modifications. With a decrease in deacetylation, the coagulation effect tends to increase [87]. In a representative work, Hu et al. [88] investigated the effects of different degrees of deacetylation on the clotting time of rabbit blood in vitro. The time it took to coagulate rabbit blood was 537 ± 10 s for the less deacetylated CS and 1035 ± 15 s for the more deacetylated CS (*p* < 0.05). Some studies reported that CS with deacetylation <80% is conducive to the formation of a network space structure [89,90]. After the low-deacetylation CS contacts the blood, the cationic molecules are more likely to combine with the negatively charged red blood cells, which improves the coagulation efficiency. CS with high deacetylation has more amino groups which form hydrogen bonds with intramolecular hydroxyl groups, resulting in a lower conductivity than CS with low deacetylation [89]. Meanwhile, deacetylation may also cause a difference in solution viscosity, and the viscosity of the low-deacetylation CS solution is higher than that of the high-deacetylation one [91]. Other studies further refined the protonation degree of CS: high deacetylation CS can be fully protonated, and a high protonation degree can inhibit the activation of platelets and the related coagulation factors. Low deacetylation CS has fewer amino groups; the protonation degree was lower than that of high-deacetylation CS after reacting with the same amount of acid. Therefore, low-deacetylation CS has a relatively suitable degree of protonation, which is beneficial to blood cell coagulation [92]. 

Moreover, the relative molecular weight also affects the coagulation-promoting effect of CS. Hattori et al. [87] investigated the effects of relative molecular weights on blood aggregation using 10 different CS species (including oligomers). The highest aggregation of erythrocytes and platelets was observed for CS at a relative molecular mass of about 8.6 kDa and a degree of deacetylation of about 85%. In contrast, CS with a high molecular weight (247 kDa) inhibited the aggregation of erythrocytes and platelets. The results indicate that the aggregation of CS is dependent on the interaction of positively charged CS with negatively charged erythrocytes, platelets, and plasma proteins. The proper balance of positive charges of CS with negative charges of blood cells and certain proteins is important. Therefore, an appropriate amount of positively charged amines is essential to stop bleeding. 

### 2.4. Effects of Chemical Modifications on Hemostasis

CS can react with many functional groups to graft or cross-link. In addition, amino groups can undergo alkylation, and quaternization, and react with aldehydes and ketones. Moreover, after introducing hydrophobic or hydrophilic groups into the polymer backbone, the stability of CS can be improved [93]. 

#### 2.4.1. N-alkyl Modification

N-alkyl modification is a typical way to introduce hydrophobic groups to the CS backbone. The hydrophobic alkyl side groups can be non-covalently inserted into the erythrocyte membrane, which significantly increases the degree of aggregation of red blood cells [94]. In addition, a certain degree of alkyl substitution and length of carbon chain on the substituent (usually between several to a dozen carbons) can significantly accelerate blood coagulation and enhance cell adhesion and hemostasis. Based on this criterion, after comparison with N-alkyl CS with different carbon chain lengths, it was found that N-octadecyl CS has a short whole-blood agglutination time and excellent hemostatic properties [95]. In another study, the experimental rats were directly injected with the hydrophobic alkyl-modified CS at the injury site, and clots were successfully formed. After 6 weeks, there was no morbidity or death, and the tissue adhesion was minimal [96]. Hydrophobically modified CS gauze was prepared by reacting CS with *N*-decanal (Schiff base reaction). The lyophilized hydrophobic alkyl-modified CS was used in a porcine arterial transection model to control bleeding from a fatal arterial injury. The results showed that the total blood loss in the hydrophobic alkyl-modified CS gauze treatment group (4.7 mL/kg) was lower than that in the control group (13.4 mL/kg). Additionally, hydrophobic alkyl-modified CS mixed with sodium citrate rapidly converted the human blood into an elastic gel. Its gelling ability was similar to that of fibrin-based sealants [97].

#### 2.4.2. Carboxyl Modification

Carboxyl modification is another typical route to adding hydrophilic groups to the CS backbone. Carboxymethyl CS (CMCS) has antibacterial, hemostasis, and healing effects, and shows an enhanced histocompatibility compared to CS. Controlling the appropriate degree of carboxymethyl substitution can increase the hydrophilicity of CS. In addition, the CMCS hydrogel helps to locally absorb the excess water and enrich the procoagulant substances in the blood, which significantly reduces the coagulation time in vitro and in vivo [98]. For instance, Wang et al. [99] prepared a novel hemostatic hydrogel by reacting the carboxyl group in CMCS with the amino group of poly(lysine). The hydrogel showed good mechanical and swelling properties with an elastic modulus of up to 4000 Pa s. In the in vitro coagulation assay, the weight of two blood samples changed from 4.0 g to 3.9 g (experimental group) and 3.1 g (control group), suggesting that the hydrogel can promote blood clotting in wounds.

#### 2.4.3. Hydroxybutyl Modification

**Figure 4:**
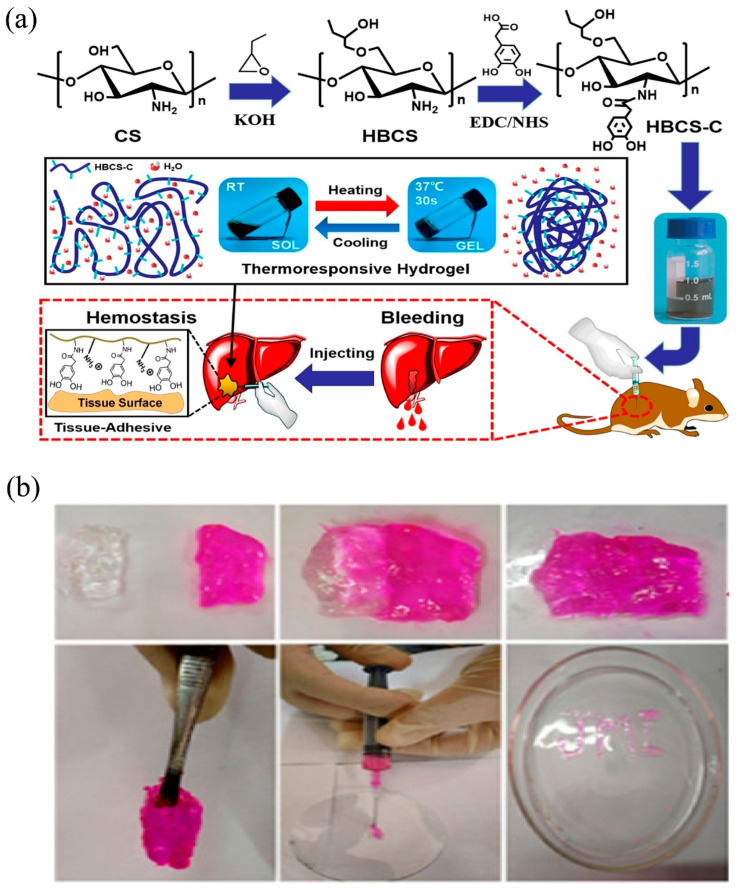
(**a**) Schematic diagram of the preparation, temperature-sensitive sol–gel transition, injection, and adhesion of HBCS-C hydrogel towards the mice liver [100]. Copyright 2020 American Chemical Society. (**b**) SCS-GAMA hydrogels were cut in half (one part unloaded and the other part loaded with Rhodamine-B) at 2 and 6 h to diffuse and self-heal, and the self-healing properties of SCS-GAMA hydrogels were confirmed by lifting the hydrogels with forceps with extruded hydrogels through a syringe [101]. Copyright 2020 American Chemical Society.

Hydroxybutyl CS can be prepared by reacting hydroxybutyl with the C-6 hydroxyl and C-2 amino groups of CS. Hydroxybutyl CS is soluble in water and is temperature sensitive. In a study, Wang and co-workers prepared catechol–hydroxybutyl CS (HBCS-C) hydrogels as potential hemostatic material. As shown in Figure 4a, HBCS-C hydrogels were prepared by grafting hydroxybutyl and catechol groups onto the CS backbone. HBCS-C hydrogels displayed excellent thermosensitive properties and exhibited good liquid gel properties at different temperatures by changing the hydrophilic–hydrophobic interactions and hydrogen bonding. Moreover, the hydrogel showed good adhesion properties, and therefore, it can strongly adhere to the tissue surface through the interaction of catechol groups and the amino groups of tissues. In order to investigate the in vivo hemostatic effect of the HBCS-C hydrogel, a rat liver hemorrhage model was constructed. The results showed that the bleeding was stopped within 30 s after injection of HBCS-C hydrogel, while the untreated wound site was still bleeding at 120 s. After the termination of the bleeding, the blood loss was 30.7 ± 11.4 mg in the HBCS-C hydrogel group and 106.5 ± 21.6 mg in the negative control group [100]. The hemostatic effect of the HBCS-C hydrogel was significantly better than that of the control group (*, *p* < 0.05; **, *p* < 0.01).

#### 2.4.4. Succinic Anhydride Modification

CS and succinic anhydride dissolved in pyridine form a solid CS-succinic acid ester (CSS) derivative. The CSS-based hemostatic agent has obvious hemostatic efficacy and safety in the rat model of liver trauma and has potential clinical application value in the treatment of severe internal bleeding by intravenous administration [102]. Pandit et al. [101] synthesized the multialdehyde modified gum arabic (GAMA), which was further used as a non-toxic cross-linking agent to develop biocompatible succinic anhydride modified CS (SCS)-based injectable hydrogels. In this study, GAMA and SCS aqueous solutions were mixed at 37 °C to obtain hydrogels by Schiff base reaction. In the absence of any external stimuli, the self-healing ability of SCS-GAMA hydrogel was examined (Figure 4b). The SCS-GAMA hydrogel was cut into halves and the first half was loaded with Rhodamine-B and the other half was left untreated. After 2 h, the dye gradually diffused into the untreated hydrogel, and after 6 h, the two hydrogels were fused into a single one. The SCS-GAMA hydrogel was loaded into a syringe and then extruded through an 18-gauge needle, which showed that the SCS-GAMA has adequate injectability. In another study, Xu et al. [103] prepared a multifunctional hydrogel with antibacterial, hemostatic, and slow-release activities by the chemical cross-linking of silk fibroin (SF) with CS, using *N*-hydroxysuccinimide as a cross-linking agent. The presence of silk fibroin improved the original porosity, water absorption, and hemostatic ability of CS dressing. The characterization results showed that the CS/SF hydrogel was characterized by a higher swelling ratio under acidic conditions and an excellent compressive mechanical property (50–130 kPa) because of the three-dimensional network structure. In addition, CS/SF hydrogel showed excellent antibacterial and hemostatic properties and was not toxic to human skin cells, showing great potential as wound dressings. 

#### 2.4.5. Other Modifications

**Figure 5:**
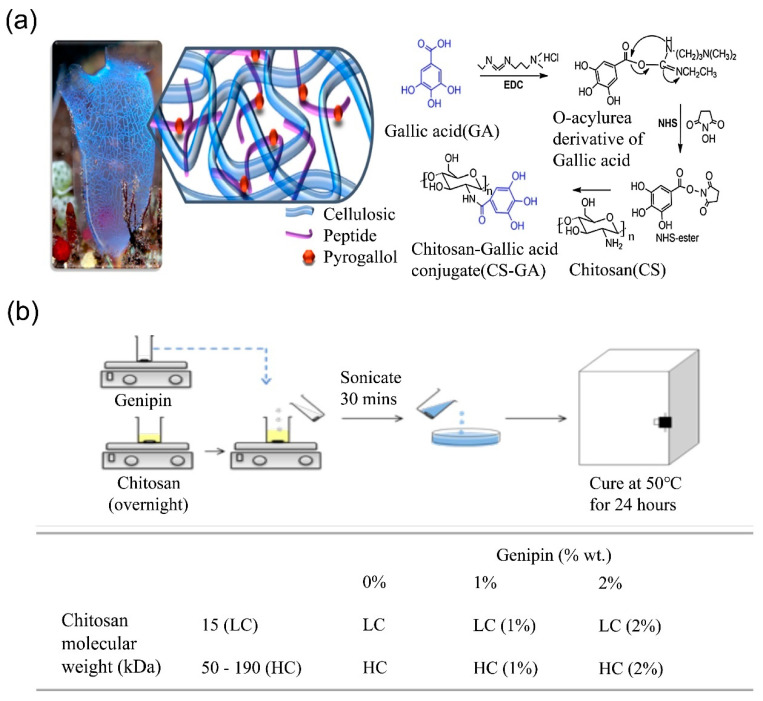
(**a**) Schematic diagram of the structure of tunicate and synthesis process of CS-GA [106]. Copyright 2020 Elsevier. (**b**) The preparation process of the CS–genipin hydrogel [109]. Copyright: 2019 American Chemical Society.

The primary amino groups of CS can be chemically modified by pyrogallol. This functional modification is beneficial in the improvement of wet adhesion, facilitating biological interface adhesion and improving hemostatic function [104]. The specific reason for this is that CS is bound to the tissue surface by electrostatic interactions of amine groups and hydrogen bonding interactions of hydroxyl groups. In addition, the partially deprotonated pyrogallol functional group can convert into a reactive aldehyde group under physiological conditions to further react with thiol and the amine groups of biological tissues and platelets [105]. Sanandiya et al. [106] synthesized a chitosan–gallic acid (CS-GA) derivative using a simple chemical modification based on the self-healing of the cysticercus. As shown in Figure 5a, gallic acid was added to the CS backbone to introduce the pyrogallol moieties by carbodiimide coupling reaction. In order to evaluate the tissue adhesive properties of a derivative, an in vitro single-lap shear adhesion assay was carried out under wet conditions and using porcine skin as the tissue substrate. The results showed that the pyrogallol moiety on the CS backbone significantly increased the solubility (50 mg/mL) and adhesion (53 kPa) properties at the physiological pH. Moreover, scanning electron microscopy showed good adhesion of CS-GA to platelets with a lower coagulation index than CS and the gauze. On the other hand, carbodiimide-guided coupling reactions lead to the formation of CS iodoacetamide derivatives, in which the iodoacetyl side group increases the hydrophobicity, and the presence of iodine improves its covalent cross-linking with proteins in plasma and blood cells. Shen et al. [107] studied the effect of the iodoacetamide substitution degree on the hemostasis of CS. The difference in the separation ratio of the citrate blood supernatant of different membranes was observed. The mean erythrocyte sedimentation ratio of a blank citrate blood sample was 33.7 min, while all other CS-based membranes resulted in a significant reduction in the erythrocyte sedimentation ratio to about half of that of the blank. Thus, the introduction of a higher iodine content may increase the cross-linking of protein with CS. CS can be modified by many substances, and this gives CS the ability to adapt to various application situations. For example, the hydrophobic and hydrophilic properties of CS could be enhanced by alkyl modification and carboxyl modification, respectively, and the wet adhesion of CS could be enhanced by succinic anhydride modification. Other specific chemical modifications which can impart different properties to CS can be found in Table 2.

**Table 2:** Construction of hemostatic hydrogels and their properties by combining different materials with CS.

Materials	Advantages	References
Catechol-functionalized CS	Superior mechanical performance and stability, strong adhesiveness, excellent hemostatic property, and injectable and thermosensitive properties	[108]
N-alkyl-functionalized CS	Hydrophobic properties, excellent hemostasis property and high adhesion strength	[95,96,97]
Carboxyl-functionalized CS	Good mechanical properties, swelling properties, antibacterial, hemostatic and pro-healing properties	[98,99]
Hydroxybutyl-functionalized CS	Thermosensitive properties, high adhesion strength and hemostatic property	[100]
Succinic anhydride-functionalized CS	Non-toxicity, hemostatic properties, and good mechanical and swelling properties	[101,102,103]
Thiol-functionalized CS	Non-toxicity, excellent hemostatic property, and hemostatic and high adhesion strength	[81]

## 3. Preparation of CS Hydrogel and Its Application as Hemostatic Materials

### 3.1. Preparation Methods of CS Hydrogel

CS hydrogels have excellent biodegradability, self-healing, swelling, and mechanical properties. These properties are partially related to the preparation methods [110], and can be exploited in wound dressings, drug release carriers, artificial skin, biosensors, etc.

#### 3.1.1. Chemical Cross-Linking

Chemically cross-linked hydrogels are primarily formed by the reaction of polymer functional groups with cross-linking agents. Cross-linking agents are compounds with two or more reactive functional groups that can cross-link with other polymers. The most commonly used cross-linking agents for the preparation of CS-based hydrogels are glutaraldehyde, formaldehyde, ethylene glycol diglycidyl acid, and genipin [111]. 

##### The Schiff Base Reaction

The Schiff reaction is a chemical reaction involving a dy-namic covalent imine bond formation via the crosslinking of amine groups and aldehyde groups [112]. Taking into consideration that human tissues have a large number of amino groups, the aldehydes or ketones reaction further facilitates the binding of hydrogels with biological tissues. In a recent study, Chen et al. [113] prepared a composite hydrogel of tetra-arm-end benzaldehyde polyethylene glycol and dodecyl-modified CS, and loaded the hydrogel with the vascular growth factor. The DCS and tetra-armed telobenzaldehyde PEG solutions were mixed at room temperature to obtain transparent hydrogel. The hydrogel exhibited injectability and high adhesion, which can repair vascular hemorrhage and liver hemorrhage. In addition, chronic wound healing in an infected total skin defect model showed that the modified CS hydrogel significantly enhanced the formation of blood vessels and granulation tissue, and promoted wound healing.

**Figure 6:**
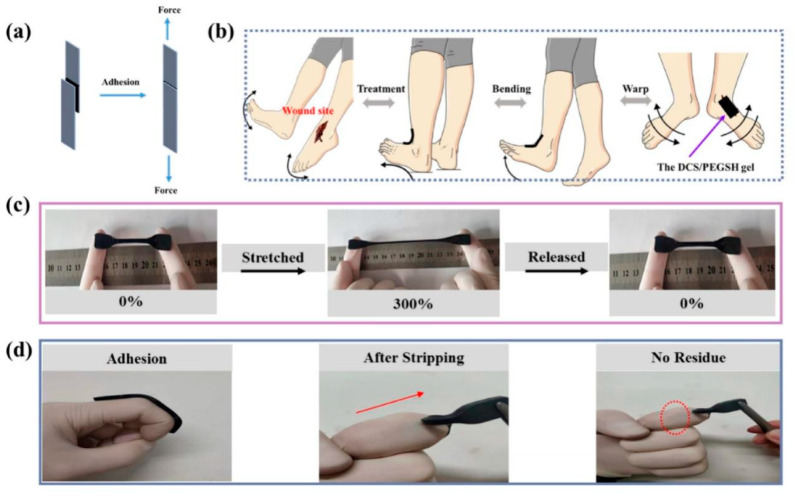
Adhesive performance of DCS/PEGSH composite hydrogel. (**a**) Schematic diagram of hydrogel adhesion and stretching. (**b**) Adhesion of hydrogel on different deformation joints. (**c**) At 300% recoverable strain, the hydrogel can adhere directly to the rubber glove without additional tape. (**d**) The rubber glove shows peeling hysteresis and no residue during peeling [114]. Copyright: 2021 Elsevier.

In a recent study, Song et al. [114] prepared a CS-based hydrogel (DCS-PEGSH) with multilevel pore structures. The 3-(3,4dihydroxyphenyl)propionic acid-modified CS (DCS) was cross-linked with p-hydroxybenzaldehyde-modified PEG sebacate (PEGSH). The fabricated hydrogel with multilevel pore structures has good cytocompatibility, proper stretchability (~780%), and blood absorption levels (1300% ± 50%). The adhesion properties of the materials were tested by cutting DCS/PEGSH hydrogels as shown in Figure 6a. DCS/PEGSH showed strong adhesion to various substrates including rubber gloves, human skin, plastic tubes, and pig skin. It was observed that when the hydrogel was attached to the knuckles, it could move freely without falling off (Figure 6b). Meanwhile, in the absence of an additional adhesive, the adhesive strength of the hydrogels (Figure 6c) was sufficient to adhere to fingers with rubber gloves with a recoverable strain of 300%. In the case of pigskin experiments, the shear strength of two pigskins joined by the hydrogel was used to evaluate its adhesion and reproducibility (Figure 6d). The reproducible adhesive performance ensured that it adhered firmly to the bleeding wound without peeling off in both static and dynamic wet environments. In hemostasis experiments, mice lost nearly 90% less blood from the liver compared to controls. Moreover, the prepared hydrogel had a shorter clotting time (50 s) and a lower clotting index (41) compared to commercial CS sponges, which has a higher clotting time (288 s) and longer clotting index (65). In a representative study, Liu et al. [115] prepared a novel injectable hemostatic hydrogel based on a Schiff base reaction. They first prepared aminocarboxymethyl CS (ACC) by grafting ethylenediamine onto carboxymethyl CS. The AHES/ACC hydrogel was fabricated by the Schiff base reaction between the aldehyde group of aldehyde hydroxyethyl starch (AHES) and the amino group of ACC. The designed AHES/ACC hydrogel exhibited good adhesion properties and can be used for hemostasis.

##### Glutaraldehyde Reaction

Glutaraldehyde can be cross-linked with CS. Thus, this chemical cross-linker has been extensively used to prepare CS hydrogels for biomedical applications. Masood et al. [116] reported a novel CS/PEG/AgNO_3_-based hydrogel by cross-linking CS with glutaraldehyde and being loaded with nano-silver to accelerate wound healing. PEG and CS solution were used to reduce silver nitrate and silver nanoparticles (AgNPs). The prepolymer was then cross-linked with glutaraldehyde to form the hydrogel. The results showed that the hydrogel has a higher porosity and better swelling than the CS-PEG hydrogel. Moreover, it can slowly and permanently release AgNPs, which have excellent properties as an efficient bactericide and antioxidant. Risbud et al. [117] prepared polyvinylpyrrolidone/β-CS semi-interpenetrating network hydrogels using glutaraldehyde as the cross-linking agent. The swelling kinetic behavior showed that the amount of glutaraldehyde cross-linking agent had an important effect on the swelling kinetics of the semi-interpenetrating network. The melting temperature of the hydrogels gradually increased with the increase in cross-linking groups. In a blood compatibility study, there was no significant change in whole–blood viscosity, plasma viscosity, and red blood cell hardness, compared with the blank control group, demonstrating that the hydrogel has good biocompatibility.

##### Other Chemical Reactions

Genipin can be used as a cross-linking agent. Under acidic and neutral conditions, it reacts spontaneously with primary amines from the polymer chain. It can also cross-link with CS and other polysaccharides to make artificial bones, wound dressings, etc. Since the aldehyde group is toxic for cells and causes severe inflammation in the human body, the low toxicity of genipin makes it a preferred cross-linker for CS hydrogel preparation. As shown in Figure 5b, Heimbuck et al. [109] used low molecular-weight CS (15 kDa) and high molecular-weight CS (50–190 kDa) to synthesize CS hydrogels, by mixing the CS solutions with genipin. Then, the precursor solution was heated uniformly in an oven at 50 °C for 24 h to ensure hydrogel formation. The non-cross-linked control CS films were synthesized in the same way in the absence of genipin. Further results showed that the CS–genipin hydrogels were able to neutralize the pH of the environment while absorbing 230% of the aqueous solution. The bacterial activity studies showed that the hydrogel was able to inhibit approximately 70% of bacterial growth while maintaining its biocompatibility on fibroblasts and keratinocytes in vitro. In addition, the CS–genipin hydrogel has a good hemostatic effect and can promote wound healing. 

#### 3.1.2. Physical Cross-Linking

Physically cross-linked hydrogels are formed by physical entanglements through hydrogen bonding, hydrophobic association or electrostatic interactions. Physically cross-linked hydrogels are reversible and can be transformed back to a solution state when external conditions changed. Moreover, due to the presence of reversible physical cross-linking bonds, physically cross-linked hydrogels often have self-healing properties. Physical cross-linking can avoid the toxicity of chemical cross-linking agents. However, the main disadvantages of physically cross-linked hydrogels are their poor mechanical properties and the difficulties in controlling their pore sizes [118]. Hydrophobic interaction is an important method to prepare physically cross-linked hydrogels. Polymers with hydrophobic regions can be cross-linked in an aqueous environment by thermal gelation, and the hydrophobic segments are grafted after polymerization or directly synthesized into block copolymers [119,120]. Geng et al. [121] prepared cellulose nanofiber/CS (CNF/CS) and acrylamide-co-acrylic acid (PAM-AA) hydrogels by polyelectrolyte complexation. The electrolyte complexation cross-linking by Fe^3+^ showed excellent mechanical properties. The average strength and elongation at the break of the PAM-AA/CNF/CS hydrogels were 11 MPa and 480%, respectively. Fan et al. [122] prepared CS/gelatin/polyvinyl alcohol (PVA) hydrogels using the γ-ray irradiation method. The tensile strength of the CS/Gel/PVA hydrogel was improved compared to the Gel/PVA hydrogel. The highest tensile strength reached 2.2 MPa. In coagulation assay experiments, all hydrogels showed good coagulation effects. The antithrombotic activity was determined by a relevant parameter called the coagulation index (BCI). In general, the lower the BCI, the better the coagulation effect of the material. When the mass ratio of CS/Gel was 1:1, the 5 min BCI reached 0.032. The hydrogel also exhibited good sensitivity, swelling ability and water evaporation rate. Therefore, the hydrogel has a promising application in wound dressing. Chemical cross-linking is an irreversible reaction, and the degree of cross-linking is stronger compared to physical cross-linking. Biocompatible cross-linking agents with better biocompatibility have greater potential to be used for CS cross-linking. Physical cross-linking is reversible, and its unique self-healing and injectable properties open up new application prospects for CS.

### 3.2. Application of CS Hydrogel as Hemostatic Materials

CS-based hydrogel has a good biocompatibility and can absorb wound exudate, avoid secondary wound infection, induce rapid wound healing, and prevent scarring. In addition, CS-based hydrogels can be loaded with bioactive substances (drugs, antigens, antibodies, growth factors, stem cells, etc.) and is slowly released at the wound site [123]; therefore, the CS-based hydrogel is a good candidate to be a hemostatic agent.

#### 3.2.1. Prerequisites as Hemostatic Hydrogels

**Figure 7:**
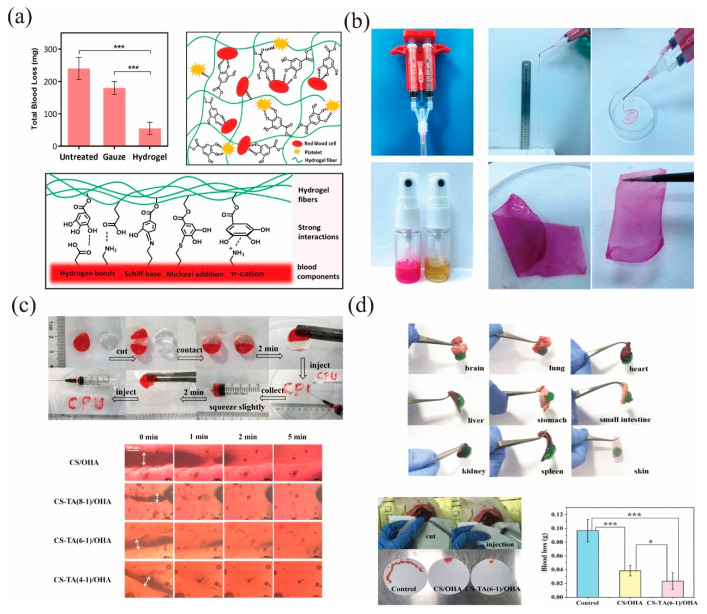
(**a**) Hydrogel covalent cross-linking principle, hydrogel-platelet interaction, comparison graph of wound bleeding after 180 s with and without adding hydrogel. (*** *p* < 0.001) (**b**) Injectable self-supporting sprayable CMCS-TA-BDBA hydrogels prepared using a double syringe [126]. Copyright 2020 American Chemical Society. (**c**) Self-healing and injectable properties. (**d**) Photographs of CS/TA-OHA adhering to organs and tissues of rats, and the amount of bleeding in rats under different treatments (* *p* < 0.05; *** *p* < 0.001.) [112]. Copyright 2022 American Chemical Society.

A CS-based hemostatic hydrogel can be injected into the body and fill an irregularly shaped defect area with minimal surgical trauma [124]. Self-healing hydrogels are hydrogels that can recover their structure and function after physical damage, either autonomously or under external stimuli [125]. These hydrogels can reduce the sudden release of drugs and prolong the lifespan of the hydrogel. For example, Geng et al. [126] designed a CMCS-based injectable and sprayable hydrogel by mixing CMCS, tannic acid (TA), and 1,4-benzene boronic acid (BDBA). The CMCS-TA-BDBA hydrogel showed a porous morphology, good biocompatibility, and self-healing properties. Due to the presence of BDBA, the hydrogel can be formed within 10 s. As shown in Figure 7a, the CMCS-TA-BDBA hydrogel has excellent hemostatic properties. By injecting the CMCS-TA-BDBA hydrogel into the bleeding site, the total bleeding volume was 55 ± 19 mg compared to 180 ± 20 and 240 ± 35 mg for the gauze-treated and blank control groups, respectively. The bleeding volume was reduced by 77% in CMCS-TA-BDBA hydrogel treatment relative to the blank control group. As shown in Figure 7b, hydrogel pieces stained in different colors can pass through a 26 G needle without being blocked and gradually heal into a whole block, indicating that the hydrogel has efficient self-healing properties. Therefore, this new injectable and aerosolized hydrogel has good application prospects in the fields of hemostasis and wound dressing. Similarly, by reacting TA-modified CS with oxidized hyaluronic acid (OHA), Liu et al. [112] prepared a composite CS hydrogel with hemostatic, adhesive, anti-inflammatory, and slow degradation properties. The CS/TA-OHA hydrogel exhibited good self-healing properties due to the presence of dynamic reversible covalent bonds and reversible hydrogen bonds. As shown in Figure 7c, the two parts of the cut hydrogel self-healed within 2 min of when they made contact with each other. The adhesion of the CS/TA-OHA hydrogel to the organs and tissues of rats is depicted in Figure 7d. An in vivo hemostasis test was performed in the rat liver injury model, and when the liver was cut and started to bleed, the hydrogel was injected into the wound to stop the bleeding. The amount of blood loss after hydrogel treatment was greatly reduced compared to the untreated control group. Further studies confirmed that the hydrogel had a good adhesive effect, and it was able to close the wound and prevent bacterial infection.

The combination of self-healing properties and hemostatic properties can be used in scenarios such as trauma hemostasis, tissue adhesion, and bone repair [127]. Tang et al. [128] used the double cross-linking of MgO-catechol and Schiff basic bonds to form hydrogels. MgO was added to catechol-modified CS (CHI-C) and oxidized dextran (ODex) to prepare biofunctional hydrogels (CCOD-MgO) for full-thickness skin wound repair. CCOD-MgO hydrogel exhibited high tissue adhesion, good self-repair, hemostatic function, and a low swelling ratio. Statistical measurements of the burn trauma area showed that the CCOD-MgO group was more efficient than the others in repairing tissue trauma. Furthermore, the in vitro antibacterial properties of CCOD-MgO were superior to CHI-C/ODex hydrogel because of the synergistic effect of CS and MgO. Feng et al. [129] designed a hemostatic CS and graphene oxide (GO) hydrogel with injectable, self-healing, and adhesive properties, by heating a mixture of CS and GO. The dynamic reversible non-covalent bonds between CS and GO conferred the abilities of injectability and self-healing to the hydrogels. Additionally, the mechanical and rheological properties of the hydrogel can be controlled by varying the amount of GO. In vivo experiments in a rat liver hemorrhage model and a total skin defect model confirmed that the CSGO hydrogel has good hemostatic and wound healing abilities. In another study, Zhang et al. [120] prepared the CS/GO hydrogel by mixing CS and GO in an alkaline solution. The results of the mechanical property tests showed that the CS/GO hydrogel has an excellent tensile strength (5.4 MPa). In addition, the CS/GO hydrogel was characterized by a pH-driven shape memory effect. Due to the pH change, the physical cross-linking through hydrophobic interactions underwent a reversible transformation and exhibited the characteristics of the shape memory effect.

#### 3.2.2. CS-Based Hemostatic Hydrogels 

**Figure 8:**
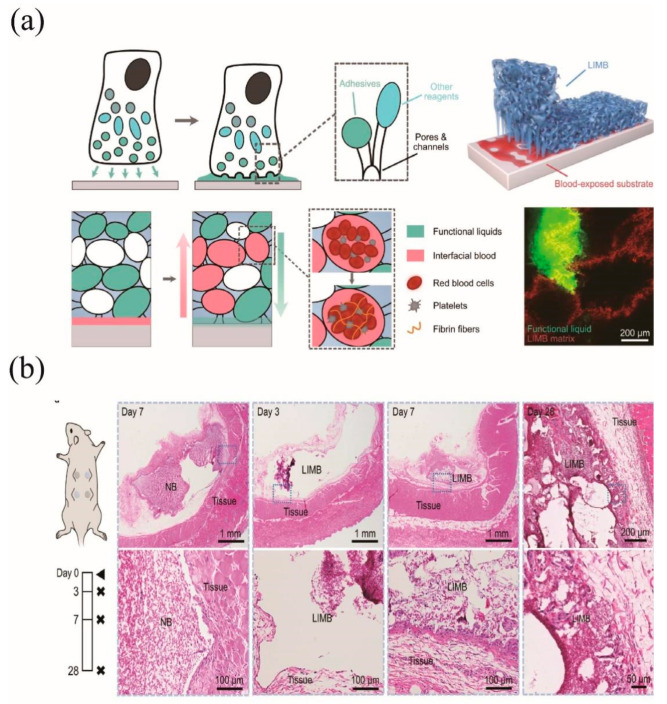
(**a**) Structure of LIMB, diagram of adherent blood and absorption of interfacial fluid. (**b**) Diagram of repair of mouse skin after burns, 0, 7, 14, and 21 days [132]. Copyright 2022.

CS hemostatic adhesives have gained widespread attention as wound dressing adhesives due to their ease of handling, non-invasive bonding, immediate sealing, and hemostatic effect [130]. Therefore, CS bioadhesives can be widely used in biomedical fields such as drug delivery, wound dressing, hemostasis and sutureless surgery [131]. For instance, Bao et al. [132] designed a liquid-infused microstructured bioadhesive (LIMBs) formed from a macroporous, tough CS dry gel infused with a functional fluid. The CS dry gel rapidly absorbed interfacial fluids and can promote blood clotting. As shown in Figure 8a, the dry hydrogels were prepared by a combination of covalently cross-linked polyacrylamide and physically cross-linked CS and were freeze-dried. Afterwards, the freeze-dried dry gels were partially infused with binder functional fluids, including CS and hydroxyl-succinimide fluids, to facilitate the formation of amide bonds with the tissue. Their synergistic effect allows this hemostatic adhesive to produce tough adhesion properties in human and porcine tissues as well as on various material surfaces, to meet the adhesion needs. The hemostasis and biocompatibility of LIMBs were significantly higher in rats and pigs compared to other commercial products. Furthermore, histological sections showed no evidence of active or chronic inflammation or allergic eosinophil response (Figure 8b), indicating that LIMB possesses good biocompatibility as a bio-adhesive and hemostatic sealant. 

Recently, Jung et al. [133] designed in situ formed CS/PEG hydrogels with high mechanical properties and adhesion using the enzymatic cross-linking reaction of horseradish peroxidase. The resulting hydrogels displayed good hemostatic ability and were highly biocompatible in vivo, which is comparable to commercially available fibrin gels. The tissue adhesive strength of the CS/PEG hydrogel was evaluated using a modified pull-shear test. Under the same compression test conditions as for CS/PEG hydrogels, all hydrogels showed enhanced adhesion to fibrin glue, with 3–13 times the strength of fibrin glue. The same trend was shown in the compressive tests, with higher compressive strength and toughness than fibrin glue being found. The CS/PEG hydrogels have not only controllable and adjustable properties, but also tissue adhesion properties, which make them potentially promising for biomedical applications.

## 4. Conclusions and Future Perspectives

With the development of science, a wide variety of hemostatic materials have emerged due to the necessity of efficient and safe repair of wounds caused in battlefields, surgical procedures, and traffic accidents. Depending on the wound type, these hemostatic materials also have different applications. For example, bleeding from external wounds caused by sharp objects, traffic accidents, battlefields, etc., can be stopped using gauze, bandages, and tourniquets. There has been a tremendous level of development and commercialization in various types of hemostatic agents in modern medicine. However, in situations of battlefields and surgical bleeding, these hemostatic materials confront many obstacles which may prevent wounds from healing. Despite sustained advances in the use of hemostatic agents in modern medicine, many obstacles limit their successful use in the treatment of uncontrolled bleeding. Therefore, the development of fast, efficient, easy-to-handle, and multifunctional hemostatic materials is of great significance and highly demanded. 

Hydrogels can be prepared using natural or synthetic polymers and have promising applications in the field of hemostatic materials. CS-based hydrogels are highly favored because of their low cost, use of renewable resources, good biocompatibility, high efficiency, and rapid hemostasis due to their abundant amino groups. Moreover, they can adsorb red blood cells and plasma proteins through electrostatic interactions to promote platelet adhesion and achieve rapid hemostasis. In addition, CS can stimulate collagen deposition and self-assembly near the wound. CS derivatives obtained by the modification of CS confer additional functions to CS while enhancing the hemostasis. In addition, CS-based hydrogels can be loaded not only with bioactive substances such as growth factors and antibiotics but also with stem cells as wound dressings for the treatment of larger skin defects. However, these hydrogels also have poor mechanical properties and it is difficult to control the gelling time. Therefore, the preparation of mechanically durable CS hemostatic hydrogels with controllable gelation behaviors constitutes a priority of future research. Moreover, the combination of homeostasis and other functionalities may also promote the development of CS-based hemostatic hydrogels [134,135]. In summary, we believe that CS-based hydrogels will revolutionize the choice of hemostatic materials in surgical and other emergency occasions and improve the treatment quality of patients with uncontrolled bleeding. 

## Figures and Tables

**Table 1:** Advantages and disadvantages of common polymers as hemostatic hydrogels.

Classification	Materials	Advantages	Limitations	References
Natural polymers	CS	Cheap and easy production, renewable resources, biocompatibility, degradability	May produce allergic reactions	[36,37,38,39]
	Alginate	Low cost, biocompatibility, low toxicity, adjustable gel properties	Poor stability under physiological conditions, low tissue adhesion, and poor mechanical properties	[40,41]
	Hyaluronic acid	Excellent glue formation, biocompatibility, biodegradability, inherent swelling properties	Limited treatment for hemorrhage, poor mechanical properties	[42,43,44]
	Gelatin	Biocompatibility, biodegradability, mild to moderate bleeding without suture fixation	Limited treatment for hemorrhage, possible allergic reactions	[45,46,47]
	Silk	High biocompatibility, biodegradability, and adjustable mechanical properties	Low adhesion properties in humid and dynamic environments	[45,48,49]
Synthetic polymers	Polyethylene glycol (PEG)	High hydration capacity, biocompatibility, non-toxicity, high structural flexibility	High expansion ratio leading to compression of nerves and limited shelf life	[50,51,52]

## References

[1] Watts S.A., Smith J.E., Woolley T., Rickard R.F., Gwyther R., Kirkman E. (2022). Resuscitation with whole blood or blood components improves survival and lessens the pathophysiological burden of trauma and haemorrhagic shock in a pre-clinical porcine model. Eur. J. Trauma Emerg. Surg..

[2] Corral M., Ferko N., Hollmann S., Broder M.S., Chang E. (2015). Health and economic outcomes associated with uncontrolled surgical bleeding: A retrospective analysis of the premier perspectives database. Clin. Outcomes Res..

[3] Malik A., Rehman F.U., Shah K.U., Naz S.S., Qaisar S. (2021). Hemostatic strategies for uncontrolled bleeding: A comprehensive update. J. Biomed. Mater. Res. Part B Appl. Biomater..

[4] Lee S.H., Yun S.J., Ryu S., Choi S.W., Kim H.J., Kang T.K., Oh S.C., Cho S.J. (2018). Massive bleeding from inferior mesenteric vein with hypovolemic shock: A rare complication of acute pancreatitis. J. Emerg. Med..

[5] Yu Y., Zheng X., Liu X., Zhao J., Wang S. (2023). Injectable carboxymethyl chitosan-based hydrogel for simultaneous anti-tumor recurrence and anti-bacterial applications. Int. J. Biol. Macromol..

[6] Chen Z., Yao J., Zhao J., Wang S. (2023). Injectable wound dressing based on carboxymethyl chitosan triple-network hydrogel for effective wound antibacterial and hemostasis. Int. J. Biol. Macromol..

[7] Cai J., Guo J., Wang S. (2023). Application of polymer hydrogels in the prevention of postoperative adhesion: A review. Gels.

[8] Pusateri A.E., Holcomb J.B., Kheirabadi B.S., Alam H.B., Wade C.E., Ryan K.L. (2006). Making sense of the preclinical literature on advanced hemostatic products. J. Trauma..

[9] Binnetoglu K., Kumandas A., Ekici H., Ozbaykus A.C. (2022). Comparison of the algan hemostatic agent with floseal in rat liver laceration bleeding model. Eurasian J. Med..

[10] An S., Jeon E.J., Jeon J., Cho S.-W. (2019). A serotonin-modified hyaluronic acid hydrogel for multifunctional hemostatic adhesives inspired by a platelet coagulation mediator. Mater. Horiz..

[11] Fonouni H., Kashfi A., Majlesara A., Stahlheber O., Konstantinidis L., Gharabaghi N., Kraus T.W., Mehrabi A., Oweira H. (2018). Hemostatic efficiency of modern topical sealants: Comparative evaluation after liver resection and splenic laceration in a swine model. J. Biomed. Mater. Res. B Appl. Biomater..

[12] Beudert M., Gutmann M., Luhmann T., Meinel L. (2022). Fibrin sealants: Challenges and solutions. ACS Biomater. Sci. Eng..

[13] Daud A., Kaur B., McClure G.R., Belley-Cote E.P., Harlock J., Crowther M., Whitlock R.P. (2020). Fibrin and thrombin sealants in vascular and cardiac surgery: A systematic review and meta-analysis. Eur. J. Vasc Endovasc. Surg..

[14] Valji K. (2014). Cyanoacrylates for embolization in gastrointestinal bleeding: How super is glue?. J. Vasc. Interv. Radiol..

[15] Zhou L., Zhao J., Chen Y., Zheng Y., Li J., Zhao J., Zhang J., Liu Y., Liu X., Wang S. (2020). MoS_2_-ALG-Fe/GOx hydrogel with Fenton catalytic activity for combined cancer photothermal, starvation, and chemodynamic therapy. Colloids Surf. B.

[16] Wang T., Yi W., Zhang Y., Wu H., Fan H., Zhao J., Wang S. (2023). Sodium alginate hydrogel containing platelet-rich plasma for wound healing. Colloids Surf. B Biointerfaces.

[17] Xie M., Zeng Y., Wu H., Wang S., Zhao J. (2022). Multifunctional carboxymethyl chitosan/oxidized dextran/sodium alginate hydrogels as dressing for hemostasis and closure of infected wounds. Int. J. Biol. Macromol..

[18] Zhang Y., Zhu C., Zhang Z., Zhao J., Yuan Y., Wang S. (2021). Oxidation triggered formation of polydopamine-modified carboxymethyl cellulose hydrogel for anti-recurrence of tumor. Colloids Surf. B.

[19] Zhu Y., Zhang Y., Wu H., Wang S., Li X. (2023). An oxidative polymerized carboxymethyl cellulose hydrogel for the combined anti-tumor recurrence. J. Mater. Sci..

[20] Liu X., Zhang Y., Wu H., Tang J., Zhou J., Zhao J., Wang S. (2023). A conductive gelatin methacrylamide hydrogel for synergistic therapy of osteosarcoma and potential bone regeneration. Int. J. Biol. Macromol..

[21] Ouyang Y., Zhao J., Wang S. (2023). Multifunctional hydrogels based on chitosan, hyaluronic acid and other biological macromolecules for the treatment of inflammatory bowel disease: A review. Int. J. Biol. Macromol..

[22] Han W., Wang S. (2023). Advances in hemostatic hydrogels that can adhere to wet surfaces. Gels.

[23] Jia Z., Zhu F., Li X., Liang Q., Zhuo Z., Huang J., Duan L., Xiong J., Wang D. (2019). Repair of osteochondral defects using injectable chitosan-based hydrogel encapsulated synovial fluid-derived mesenchymal stem cells in a rabbit model. Mater. Sci. Eng. C Mater. Biol. Appl..

[24] Jin R., Xu J., Duan L., Gao G. (2021). Chitosan-driven skin-attachable hydrogel sensors toward human motion and physiological signal monitoring. Carbohydr. Polym..

[25] Ding H., Li B., Liu Z., Liu G., Pu S., Feng Y., Jia D., Zhou Y. (2021). Nonswelling injectable chitosan hydrogel via UV crosslinking induced hydrophobic effect for minimally invasive tissue engineering. Carbohydr. Polym..

[26] Dong X., Li C., Zhang M., Zhao Y., Zhao Z., Li W., Zhang X. (2022). Multifunctional injectable hydrogel for effective promotion of cartilage regeneration and protection against osteoarthritis: Combined chondroinductive, antioxidative and anti-in fl ammatory strategy. Sci. Technol. Adv. Mater..

[27] Yan X., Fang W.W., Xue J., Sun T.C., Dong L., Zha Z., Qian H., Song Y.H., Zhang M., Gong X. (2019). Thermoresponsive in situ forming hydrogel with sol-gel irreversibility for effective methicillin-resistant staphylococcus aureus infected wound healing. ACS Nano.

[28] Liang Y., Li Z., Huang Y., Yu R., Guo B. (2021). Dual-dynamic-bond cross-linked antibacterial adhesive hydrogel sealants with on-demand removability for post-wound-closure and infected wound healing. ACS Nano.

[29] Jones M., Kujundzic M., John S., Bismarck A. (2020). Crab vs. Mushroom: A review of crustacean and fungal chitin in wound treatment. Mar. Drugs.

[30] Negm N.A., Hefni H.H.H., Abd-Elaal A.A.A., Badr E.A., Abou Kana M.T.H. (2020). Advancement on modification of chitosan biopolymer and its potential applications. Int. J. Biol. Macromol..

[31] Bakshi P.S., Selvakumar D., Kadirvelu K., Kumar N.S. (2020). Chitosan as an environment friendly biomaterial—A review on recent modifications and applications. Int. J. Biol. Macromol..

[32] Cao J., Xiao L., Shi X. (2019). Injectable drug-loaded polysaccharide hybrid hydrogels for hemostasis. RSC Adv..

[33] Xia Y.L., Yang R.H., Wang H.Y., Li Y.H., Fu C.F. (2022). Application of chitosan-based materials in surgical or postoperative hemostasis. Front. Mater..

[34] Hu B.J., Bao G.C., Xu X.X., Yang K. (2022). Topical hemostatic materials for coagulopathy. J. Mater. Chem B.

[35] Simpson A., Shukla A., Brown A.C. (2022). Biomaterials for hemostasis. Annu. Rev. Biomed. Eng..

[36] Hu Z., Zhang D.Y., Lu S.T., Li P.W., Li S.D. (2018). Chitosan-based composite materials for prospective hemostatic applications. Mar. Drugs.

[37] Lestari W., Yusry W., Haris M.S., Jaswir I., Idrus E. (2020). A glimpse on the function of chitosan as a dental hemostatic agent. Jpn. Dent. Sci. Rev..

[38] Ranjith R., Balraj S., Ganesh J., John Milton M.C. (2019). Therapeutic agents loaded chitosan-based nanofibrous mats as potential wound dressings: A review. Mater. Today Chem..

[39] Whang H.S., Kirsch W., Zhu Y.H., Yang C.Z., Hudson S.M. (2005). Hemostatic agents derived from chitin and chitosan. J. Macromol. Sci. Polym. Rev..

[40] Xie Y., Gao P., He F., Zhang C. (2022). Application of alginate-based hydrogels in hemostasis. Gels.

[41] Wang L., Hao F., Tian S., Dong H., Nie J., Ma G. (2022). Targeting polysaccharides such as chitosan, cellulose, alginate and starch for designing hemostatic dressings. Carbohydr. Polym..

[42] Chen Y., Wu L., Li P., Hao X., Yang X., Xi G., Liu W., Feng Y., He H., Shi C. (2020). Polysaccharide based hemostatic strategy for ultrarapid hemostasis. Macromol. Biosci..

[43] Wang Y., Liu G., Wu L., Qu H., Song D., Huang H., Wu C., Xu M. (2020). Rational design of porous starch/hyaluronic acid composites for hemostasis. Int. J. Biol. Macromol..

[44] Weng H., Jia W., Li M., Chen Z. (2022). New injectable chitosan-hyaluronic acid based hydrogels for hemostasis and wound healing. Carbohydr. Polym..

[45] Ndlovu S.P., Ngece K., Alven S., Aderibigbe B.A. (2021). Gelatin-based hybrid scaffolds: Promising wound dressings. Polymers.

[46] Lied G.A., Lund K.B., Storaas T. (2019). Intraoperative anaphylaxis to gelatin-based hemostatic agents: A case report. J. Asthma Allergy.

[47] Kang K., Liu Y., Song X., Xu L., Zhang W., Jiao Y., Zhao Y. (2022). Hemostatic performance of a-chitin/gelatin composite sponges with directional pore structure. Macromol. Biosci..

[48] Biswas S., Bhunia B.K., Janani G., Mandal B.B. (2022). Silk fibroin based formulations as potential hemostatic agents. ACS Biomater. Sci. Eng..

[49] Shefa A.A., Taz M., Lee S.Y., Lee B.T. (2019). Enhancement of hemostatic property of plant derived oxidized nanocellulose-silk fibroin based scaffolds by thrombin loading. Carbohydr. Polym..

[50] Okata S., Hoshina K., Hanada K., Kamata H., Fujisawa A., Yoshikawa Y., Sakai T. (2022). Hemostatic capability of a novel tetra-polyethylene glycol hydrogel. Ann. Vasc. Surg..

[51] Boerman M.A., Roozen E., Sanchez-Fernandez M.J., Keereweer A.R., Felix Lanao R.P., Bender J., Hoogenboom R., Leeuwenburgh S.C., Jansen J.A., Van Goor H. (2017). Next generation hemostatic materials based on NHS-ester functionalized poly(2-oxazoline)s. Biomacromolecules.

[52] Dhandapani V., Ringuette V., Desrochers M., Sirois M., Vermette P. (2022). Composition, host responses and clinical applications of bioadhesives. J. Biomed. Mater. Res. B Appl. Biomater..

[53] Kerris E.W.J., Hoptay C., Calderon T., Freishtat R.J. (2020). Platelets and platelet extracellular vesicles in hemostasis and sepsis. J. Investig. Med..

[54] Caspers M., Maegele M., Frohlich M. (2018). Current strategies for hemostatic control in acute trauma hemorrhage and trauma-induced coagulopathy. Expert Rev. Hematol..

[55] Leonardi M.J. (2019). Laboratory evaluation of hemostasis disorders. Physician Assist. Clin..

[56] Sundaram M.N., Mony U., Varma P.K., Rangasamy J. (2021). Vasoconstrictor and coagulation activator entrapped chitosan based composite hydrogel for rapid bleeding control. Carbohydr. Polym..

[57] Lippi G., Favaloro E.J. (2018). Laboratory hemostasis: From biology to the bench. Clin. Chem. Lab. Med..

[58] Toonstra C., Hu Y., Zhang H. (2019). Deciphering the roles of N-glycans on collagen-platelet interactions. J. Proteome Res..

[59] Zhang B., Hu X., Wang H., Wang R., Sun Z., Tan X., Liu S., Wang H. (2020). Effects of a dammarane-type saponin, ginsenoside Rd, in nicotine-induced vascular endothelial injury. Phytomedicine.

[60] Yang X., Liu W., Li N., Wang M., Liang B., Ullah I., Neve A.L., Feng Y., Chen H., Shi C. (2017). Design and development of polysaccharide hemostatic materials and their hemostatic mechanism. Biomater. Sci..

[61] Wheeler A.P., Gailani D. (2016). The intrinsic pathway of coagulation as a target for antithrombotic therapy. Hematol. Oncol. Clin. N. Am..

[62] Zhong Y., Hu H., Min N., Wei Y., Li X., Li X. (2021). Application and outlook of topical hemostatic materials: A narrative review. Ann. Transl. Med..

[63] Versteeg H.H., Heemskerk J.W., Levi M., Reitsma P.H. (2013). New fundamentals in hemostasis. Physiol. Rev..

[64] Ebhodaghe S.O. (2022). A short review on chitosan and gelatin-based hydrogel composite polymers for wound healing. J. Biomater. Sci. Polym. Ed..

[65] Hao R., Peng X., Zhang Y., Chen J., Wang T., Wang W., Zhao Y., Fan X., Chen C., Xu H. (2020). Rapid hemostasis resulting from the synergism of self-assembling short peptide and o-carboxymethyl chitosan. ACS Appl. Mater. Interfaces.

[66] Hao Y., Zhao W., Zhang L., Zeng X., Sun Z., Zhang D., Shen P., Li Z., Han Y., Li P. (2020). Bio-multifunctional alginate/chitosan/fucoidan sponges with enhanced angiogenesis and hair follicle regeneration for promoting full-thickness wound healing. Mater. Des..

[67] Wang Y.W., Liu C.C., Cherng J.H., Lin C.S., Chang S.J., Hong Z.J., Liu C.C., Chiu Y.K., Hsu S.D., Chang A.H. (2019). Biological effects of chitosan-based dressing on hemostasis mechanism. Polymers.

[68] Mukhtar M., Csaba N., Robla S., Varela-Calvino R., Nagy A., Burian K., Kokai D., Ambrus R. (2022). Dry powder comprised of isoniazid-loaded nanoparticles of hyaluronic acid in conjugation with mannose-anchored chitosan for macrophage-targeted pulmonary administration in tuberculosis. Pharmaceutics.

[69] Liu Y., Niu H., Wang C., Yang X., Li W., Zhang Y., Ma X., Xu Y., Zheng P., Wang J. (2022). Bio-inspired, bio-degradable adenosine 5’-diphosphate-modified hyaluronic acid coordinated hydrophobic undecanal-modified chitosan for hemostasis and wound healing. Bioact. Mater..

[70] Huang Y., Zhang Y., Feng L., He L., Guo R., Xue W. (2018). Synthesis of N-alkylated chitosan and its interactions with blood. Artif. Cells Nanomed. Biotechnol..

[71] Chen K.Y., Lin T.H., Yang C.Y., Kuo Y.W., Lei U. (2018). Mechanics for the adhesion and aggregation of red blood cells on chitosan. J. Mech..

[72] Chou T.-C., Fu E., Wu C.-J., Yeh J.-H. (2003). Chitosan enhances platelet adhesion and aggregation. Biochem. Biophys. Res. Commun..

[73] Shen E., Chou T., Gau C. (2006). Releasing growth factors from activated human platelets after chitosan stimulation: A possible bio-material for platelet-rich plasma preparation. Clin. Oral Implant. Res..

[74] Patil G., Pawar R., Jadhav S., Ghormade V. (2022). A chitosan based multimodal “soft” hydrogel for rapid hemostasis of non-compressible hemorrhages and its mode of action. Carbohydr. Polym. Technol. Appl..

[75] Chen L., Tianqing L. (2008). Interaction behaviors between chitosan and hemoglobin. Int. J. Biol. Macromol..

[76] Yin M., Wang Y., Zhang Y., Ren X., Qiu Y., Huang T.S. (2020). Novel quaternarized N-halamine chitosan and polyvinyl alcohol nanofibrous membranes as hemostatic materials with excellent antibacterial properties. Carbohydr. Polym..

[77] Zhou M., Liao J., Li G., Yu Z., Xie D., Zhou H., Wang F., Ren Y., Xu R., Dai Y. (2022). Expandable carboxymethyl chitosan/cellulose nanofiber composite sponge for traumatic hemostasis. Carbohydr. Polym..

[78] Thao N.T.T., Wijerathna H., Kumar R.S., Choi D., Dananjaya S.H.S., Attanayake A.P. (2021). Preparation and characterization of succinyl chitosan and succinyl chitosan nanoparticle film: In vitro and in vivo evaluation of wound healing activity. Int. J. Biol. Macromol..

[79] Sperling C., Maitz M.F., Grasso S., Werner C., Kanse S.M. (2017). A positively charged surface triggers coagulation activation through factor vii activating protease (FSAP). ACS Appl. Mater. Interfaces.

[80] Kedzierska M., Blilid S., Milowska K., Kolodziejczyk-Czepas J., Katir N., Lahcini M., El Kadib A., Bryszewska M. (2021). Insight into factors influencing wound healing using phosphorylated cellulose-filled-chitosan nanocomposite films. Int. J. Mol. Sci..

[81] Nie W., Yuan X., Zhao J., Zhou Y., Bao H. (2013). Rapidly in situ forming chitosan/epsilon-polylysine hydrogels for adhesive sealants and hemostatic materials. Carbohydr. Polym..

[82] Drozd N.N., Lunkov A.P., Il’ina A.V., Varlamov V.P. (2020). Hemocompatibility of silver nanoparticles based on conjugate of quaternized chitosan with gallic acid in in vitro experiments. Bull. Exp. Biol. Med..

[83] von Petersdorff-Campen K., Schmid Daners M. (2022). Hemolysis testing in vitro: A review of challenges and potential improvements. ASAIO J..

[84] Liu X.L., Yuan L., Li D., Tang Z.C., Wang Y.W., Chen G.J., Chen H., Brash J.L. (2014). Blood compatible materials: State of the art. J. Mater. Chem. B.

[85] Sen V.D., Sokolova E.M., Neshev N.I., Kulikov A.V., Pliss E.M. (2017). Low molecular chitosan–(poly)nitroxides: Synthesis and evaluation as antioxidants on free radical-induced erythrocyte hemolysis. React. Funct. Polym..

[86] Chen Y., Zhang Y., Wang F., Meng W., Yang X., Li P., Jiang J., Tan H., Zheng Y. (2016). Preparation of porous carboxymethyl chitosan grafted poly (acrylic acid) superabsorbent by solvent precipitation and its application as a hemostatic wound dressing. Mater. Sci. Eng. C Mater. Biol. Appl..

[87] Hattori H., Ishihara M. (2015). Changes in blood aggregation with differences in molecular weight and degree of deacetylation of chitosan. Biomed. Mater..

[88] Hu Z., Lu S., Cheng Y., Kong S., Li S., Li C., Yang L. (2018). Investigation of the effects of molecular parameters on the hemostatic properties of chitosan. Molecules.

[89] Wu J., Zhang L. (2019). Dissolution behavior and conformation change of chitosan in concentrated chitosan hydrochloric acid solution and comparison with dilute and semidilute solutions. Int. J. Biol. Macromol..

[90] Song X., Zhao Y., Liu Y., Zhang W., Yuan X., Xu L., Zhang J. (2021). Effects of degree of deacetylation on hemostatic performance of partially deacetylated chitin sponges. Carbohydr. Polym..

[91] Markushin S.G., Akopova I.I., Blagodatskikh I.V., Kulikov S.N., Bezrodnykh E.A., Muranov A.V., Yamskov I.A., Tikhonov V.E. (2018). Effect of molecular weight and degree of acetylation on adjuvantive properties of chitosan derivatives. Appl. Biochem. Microbiol..

[92] Naveed M., Phil L., Sohail M., Hasnat M., Baig M., Ihsan A.U., Shumzaid M., Kakar M.U., Mehmood Khan T., Akabar M.D. (2019). Chitosan oligosaccharide (COS): An overview. Int. J. Biol. Macromol..

[93] Varlamov V.P., Il’ina A.V., Shagdarova B.T., Lunkov A.P., Mysyakina I.S. (2020). Chitin/chitosan and its derivatives: Fundamental problems and practical approaches. Biochemistry.

[94] Verma C., Quraishi M.A., Alfantazi A., Rhee K.Y. (2021). Corrosion inhibition potential of chitosan based Schiff bases: Design, performance and applications. Int. J. Biol. Macromol..

[95] Wang X., Guan J., Zhuang X., Li Z., Huang S., Yang J., Liu C., Li F., Tian F., Wu J. (2018). Exploration of blood coagulation of N-alkyl chitosan nanofiber membrane in vitro. Biomacromolecules.

[96] Logun M.T., Dowling M.B., Raghavan S.R., Wallace M.L., Schmiedt C., Stice S., Karumbaiah L. (2019). Expanding hydrophobically modified chitosan foam for internal surgical hemostasis: Safety evaluation in a murine model. J. Surg. Res..

[97] Chaturvedi A., Dowling M.B., Gustin J.P., Scalea T.M., Raghavan S.R., Pasley J.D., Narayan M. (2017). Hydrophobically modified chitosan gauze: A novel topical hemostat. J. Surg. Res..

[98] Liu L., Lv Q., Zhang Q., Zhu H., Liu W., Deng G., Wu Y., Shi C., Li H., Li L. (2017). Preparation of carboxymethyl chitosan microspheres and their application in hemostasis. Disaster Med. Public Health Prep..

[99] Wang Y., Cao H., Wang X. (2020). Synthesis and characterization of an injectable ε-polylysine/carboxymethyl chitosan hydrogel used in medical application. Mater. Chem. Phys..

[100] Shou Y., Zhang J., Yan S., Xia P., Xu P., Li G., Zhang K., Yin J. (2020). Thermoresponsive chitosan/dopa-based hydrogel as an injectable therapy approach for tissue-adhesion and hemostasis. ACS Biomater. Sci. Eng..

[101] Pandit A.H., Mazumdar N., Imtiyaz K., Alam Rizvi M.M., Ahmad S. (2020). Self-healing and injectable hydrogels for anticancer drug delivery: A study with multialdehyde gum arabic and succinic anhydride chitosan. ACS Appl. Bio Mater..

[102] Zhang P., Li S., Zhang S., Zhang X., Wan L., Yun Z., Ji S., Gong F., Huang M., Wang L. (2018). GRGDS-functionalized chitosan nanoparticles as a potential intravenous hemostat for traumatic hemorrhage control in an animal model. Nanomedicine.

[103] Xu Z., Chen T., Zhang K.Q., Meng K., Zhao H. (2021). Silk fibroin/chitosan hydrogel with antibacterial, hemostatic and sustained drug-release activities. Polym. Int..

[104] Ju J., Jin S.B., Kim S., Choi J.H., Lee H.A., Son D., Lee H., Shin M. (2022). Addressing the shortcomings of polyphenol-derived adhesives: Achievement of long shelf life for effective hemostasis. ACS Appl. Mater. Interfaces.

[105] Tang L.L., Dang Y., Wang Y., Zhang Y.L., Hu T.S., Ding C.C., Wu H., Ni Y.H., Chen L.H., Huang L.L. (2022). Rapid fabrication of bionic pyrogallol-based self-adhesive hydrogel with mechanically tunable, self-healing, antibacterial, wound healing, and hemostatic properties. Biomater. Adv..

[106] Sanandiya N.D., Lee S., Rho S., Lee H., Kim I.S., Hwang D.S. (2019). Tunichrome-inspired pyrogallol functionalized chitosan for tissue adhesion and hemostasis. Carbohydr Polym..

[107] Shen J.L., Nada A.A., Abou-Zeid N.Y., Hudson S.M. (2020). Synthesis of chitosan iodoacetamides via carbodiimide coupling reaction: Effect of degree of substitution on the hemostatic properties. Carbohydr. Polym..

[108] Ryu J.H., Lee Y., Kong W.H., Kim T.G., Park T.G., Lee H. (2011). Catechol-functionalized chitosan/pluronic hydrogels for tissue adhesives and hemostatic materials. Biomacromolecules.

[109] Heimbuck A.M., Priddy-Arrington T.R., Padgett M.L., Llamas C.B., Barnett H.H., Bunnell B.A., Caldorera-Moore M.E. (2019). Development of responsive chitosan-genipin hydrogels for the treatment of wounds. ACS Appl. Bio Mater..

[110] Chandra Hembram K., Prabha S., Chandra R., Ahmed B., Nimesh S. (2016). Advances in preparation and characterization of chitosan nanoparticles for therapeutics. Artif. Cells Nanomed. Biotechnol..

[111] Parhi R. (2017). Cross-linked hydrogel for pharmaceutical applications: A review. Adv. Pharm. Bull..

[112] Liu S., Jiang N., Chi Y., Peng Q., Dai G., Qian L., Xu K., Zhong W., Yue W. (2022). Injectable and self-healing hydrogel based on chitosan-tannic acid and oxidized hyaluronic acid for wound healing. ACS Biomater. Sci. Eng..

[113] Chen G., Yu Y., Wu X., Wang G., Ren J., Zhao Y. (2018). Bioinspired multifunctional hybrid hydrogel promotes wound healing. Adv. Funct. Mater..

[114] Song F., Kong Y., Shao C., Cheng Y., Lu J., Tao Y., Du J., Wang H. (2021). Chitosan-based multifunctional flexible hemostatic bio-hydrogel. Acta Biomater..

[115] Liu J., Li J., Yu F., Zhao Y.X., Mo X.M., Pan J.F. (2020). In situ forming hydrogel of natural polysaccharides through Schiff base reaction for soft tissue adhesive and hemostasis. Int. J. Biol. Macromol..

[116] Masood N., Ahmed R., Tariq M., Ahmed Z., Masoud M.S., Ali I., Asghar R., Andleeb A., Hasan A. (2019). Silver nanoparticle impregnated chitosan-PEG hydrogel enhances wound healing in diabetes induced rabbits. Int. J. Pharm..

[117] Risbud M.V., Bhat S.V. (2001). Properties of polyvinyl pyrrolidone/beta-chitosan hydrogel membranes and their biocompatibility evaluation by haemorheological method. J. Mater. Sci. Mater. Med..

[118] Sanchez-Cid P., Jimenez-Rosado M., Romero A., Perez-Puyana V. (2022). Novel trends in hydrogel development for biomedical applications: A review. Polymers.

[119] Li C., Obireddy S.R., Lai W.F. (2021). Preparation and use of nanogels as carriers of drugs. Drug Deliv..

[120] Zhang Y., Zhang M., Jiang H., Shi J., Li F., Xia Y., Zhang G., Li H. (2017). Bio-inspired layered chitosan/graphene oxide nanocomposite hydrogels with high strength and pH-driven shape memory effect. Carbohydr. Polym..

[121] Geng L., Hu S., Cui M., Wu J., Huang A., Shi S., Peng X. (2021). Muscle-inspired double-network hydrogels with robust mechanical property, biocompatibility and ionic conductivity. Carbohydr. Polym..

[122] Fan L.H., Yang H., Yang J., Peng M., Hu J. (2016). Preparation and characterization of chitosan/gelatin/PVA hydrogel for wound dressings. Carbohydr. Polym..

[123] Do N.H.N., Truong Q.T., Le P.K., Ha A.C. (2022). Recent developments in chitosan hydrogels carrying natural bioactive compounds. Carbohydr. Polym..

[124] Pourshahrestani S., Zeimaran E., Kadri N.A., Mutlu N., Boccaccini A.R. (2020). Polymeric hydrogel systems as emerging biomaterial platforms to enable hemostasis and wound healing. Adv. Healthc. Mater..

[125] Tu Y., Chen N., Li C., Liu H., Zhu R., Chen S., Xiao Q., Liu J., Ramakrishna S., He L. (2019). Advances in injectable self-healing biomedical hydrogels. Acta Biomater..

[126] Geng H., Dai Q., Sun H., Zhuang L., Song A., Caruso F., Hao J., Cui J. (2020). Injectable and sprayable polyphenol-based hydrogels for controlling hemostasis. ACS Appl. Bio Mater..

[127] Ou Y., Tian M. (2021). Advances in multifunctional chitosan-based self-healing hydrogels for biomedical applications. J. Mater. Chem B.

[128] Tang X., Wang X., Sun Y., Zhao L., Li D., Zhang J., Sun H., Yang B. (2021). Magnesium oxide-assisted dual-cross-linking bio-multifunctional hydrogels for wound repair during full-thickness skin injuries. Adv. Funct. Mater..

[129] Feng W., Wang Z. (2022). Shear-thinning and self-healing chitosan-graphene oxide hydrogel for hemostasis and wound healing. Carbohydr. Polym..

[130] Li D., Chen J., Wang X., Zhang M., Li C., Zhou J. (2020). Recent advances on synthetic and polysaccharide adhesives for biological hemostatic applications. Front. Bioeng. Biotechnol..

[131] Hamedi H., Moradi S., Hudson S.M., Tonelli A.E., King M.W. (2022). Chitosan based bioadhesives for biomedical applications: A review. Carbohydr. Polym..

[132] Bao G., Gao Q., Cau M., Ali-Mohamad N., Strong M., Jiang S., Yang Z., Valiei A., Ma Z., Amabili M. (2022). Liquid-infused microstructured bioadhesives halt non-compressible hemorrhage. Nat. Commun..

[133] Jung H.Y., Le Thi P., HwangBo K.H., Bae J.W., Park K.D. (2021). Tunable and high tissue adhesive properties of injectable chitosan based hydrogels through polymer architecture modulation. Carbohydr. Polym..

[134] Zhang Z., Zhao J., Chen Z., Wu H., Wang S. (2023). A molybdenum-based nanoplatform with multienzymes mimic capacity for oxidative stress-induced acute liver injury treatment. Inorg. Chem. Front..

[135] Yang X., Wang S., Zhang X., Ye C., Wang S., An X. (2022). Development of PVA-based microsphere as a potential embolization agent. Mater. Sci. Eng. C.

